# Exploring necroptosis: mechanistic analysis and antitumor potential of nanomaterials

**DOI:** 10.1038/s41420-025-02423-x

**Published:** 2025-04-29

**Authors:** Jiaheng Dong, Jiale Zhang, Kunhou Yao, Xiao Xu, Yaqi Zhou, Lei Zhang, Changjiang Qin

**Affiliations:** 1 School of Basic Medical Sciences, Henan University, Kaifeng, 475004 China; 2 School of Life Sciences, Xiamen University, Xiamen, 361005 China; 3 Department of General Surgery, Huaihe Hospital of Henan University, Kaifeng, 475004 China

**Keywords:** Cancer therapy, Nanobiotechnology, Necroptosis

## Abstract

Necroptosis, a non-apoptotic mode of programmed cell death, is characterized by the disintegration of the plasma membrane, ultimately leading to cell perforation and rupture. Recent studies have disclosed the mechanism of necroptosis and its intimate link with nanomaterials. Nanomedicine represents a novel approach in the development of therapeutic agents utilizing nanomaterials to treat a range of cancers with high efficacy. This article provides an overview of the primary mechanism behind necroptosis, the current research progress in nanomaterials, their potential use in various diseases—notably cancer, safety precautions, and prospects. The goal is to aid in the development of nanomaterials for cancer treatment.

## Facts

- Necroptosis, as a typical form of cell death, is being increasingly explored by researchers to investigate its association with various diseases and the feasibility of its treatment.
- Multiple molecules, such as TNFR1, RIPK1, and RIPK3, play crucial roles in cellular necroptosis.
- Cellular necroptosis involves various regulatory mechanisms, including ubiquitination modification, phosphorylation modification, and MLKL regulation.
- Necroptosis exhibits a dual effect of promoting and inhibiting tumor growth, and through the study of necroptosis, it may eventually lead to anti-tumor outcomes.
- Nanomaterials can induce cellular necroptosis through multiple pathways, such as directly promoting necroptosis in cells or coupling with drugs and combining with ultrasound and phototherapy to promote necroptosis of cancer cells.

## Open questions

- What are the specific mechanisms of TNFR1, RIPK1 and RIPK3 on cell necroptosis?
- How do ubiquitination, phosphorylation, and MLKL regulate necroptosis?
- What are the implications of necroptosis for the treatment of cancer?
- How do nanomaterials exert their therapeutic effects through the necroptosis pathway?

## Introduction

Beginning in the mid-nineteenth century, Virchow et al. found that cell death by necrosis could induce illnesses. A century later, apoptosis was identified as a gene-regulated, programmed cell death distinct from necrosis [1]. It is triggered by physiological states or external stimuli and involves processes like intestinal epithelial cell renewal, T cell homeostasis, and responses to irreversible DNA damage. Apoptosis is characterized by cell shrinkage, membrane swelling, chromatin fragmentation, and aggregation [2]. In contrast, necrosis features explosive discharge of cellular contents, mitochondrial swelling, and cytoplasmic membrane breakdown [3]. Previously, necrosis was thought to be uncontrolled, but research revealed necroptosis, a regulated death pathway mediated by death receptors, RNA, DNA sensors, and other mediators [4]. It is regulated by RIPK1, RIPK3, and MLKL and can cause severe inflammation linked to tumors, inflammatory bowel disease, and liver damage [5–8]. Non-apoptotic programmed cell death, such as necroptosis, was first observed by Dominique Vercammen et al. when apoptosis inhibitors caused necrotic morphology in L929 cells stimulated by TNF-α [9]. This mode, regulated by RIP1 and RIP3, is termed “necroptosis” or “programmed necrosis.” [10]. Recently, nanomaterials have gained attention for their potential to induce necroptosis in cancer therapy. They can trigger RIPK3-independent necroptosis or induce energy shifts towards necroptosis [11, 12]. However, their use in cancer treatment faces challenges and safety concerns [13]. Further research and clinical trials are needed to confirm their safety and efficacy. This review summarizes necroptosis mechanisms and nanomaterial approaches for cancer therapy.

## Characteristics and regulatory mechanisms of necroptosis

Necrosis is divided into regulated necroptosis and unregulated necrosis. Necroptosis, regulated by RIPK1, RIPK3, and MLKL, shares morphological features with necrosis, including changes in cell membrane permeability, cell and organelle swelling, cytoplasmic membrane rupture, and release of cellular contents [6, 7]. This release can expose damage-associated molecular patterns (DAMPs) and trigger a strong inflammatory response [14]. Many stimuli can cause necroptosis, such as tumor necrosis factor (TNF) family including TNF-α, FasL and TNF-related apoptosis inducing ligand (TRAIL) [15]. And pattern recognition receptor (PRR) including TLR3, TLR4 and Z-DNA binding protein 1 (ZBP1) [16]. The TNF-α/TNFR1 pathway regulating necroptosis is relatively well understood. TNF-α binds to TNFR1, TNF-α receptor-associated death domain protein (TRADD), RIPK1 and cellular inhibitor of apoptosis protein (cIAP) 1. Cylindromatosis (CYLD) and TNFR-associated factor (TRAF) 2/5 aggregate to form membrane-associated complex I. When cIAP induces RIPK1 ubiquitination, it inhibits the formation of complex IIa and complex IIb (also known as the necrosome), thereby blocking RIPK1-mediated apoptosis or necroptosis [17]. Complex IIa is composed of molecules such as caspase-8, Fas-associated with death domain protein (FADD) and RIPK1. Complex IIb consists of caspase-8, FADD, RIPK1, RIPK3 and MLKL. Deubiquitylation of RIPK1 by CYLD can promote the assembly of complex IIa and complex IIb. CYLD deubiquitylates RIPK1, promoting necrosome assembly [18]. Caspase-8 activation in complex IIa cleaves RIPK1 and RIPK3, blocking necroptosis and activating apoptosis. Inhibition of caspase-8 activity allows RIPK1 and RIPK3 to form complex IIb, leading to MLKL phosphorylation, oligomerization, and membrane localization, ultimately causing cell membrane rupture [19]. Thus, caspase-8 activation and RIPK1/RIPK3 interaction act as a “switch” for apoptosis or necroptosis [20, 21].

Recently, it has been found that by stimulating the DNA receptor DAI (a DNA-dependent activator of interferon regulatory factor), or activating the death receptor (TNF-α activating TNFR1), FasL, TRAIL, Toll-like receptors (including TRL3 and TRL4, etc.), T-cell antigen receptor, interferon receptor, or virus infection can cause necroptosis [22–24]. Among these, necroptosis mediated by the interaction between TNF-α and TNFR1 has attracted the most attention.

### Role of TNFR1 in necroptosis

**Fig. 1:**
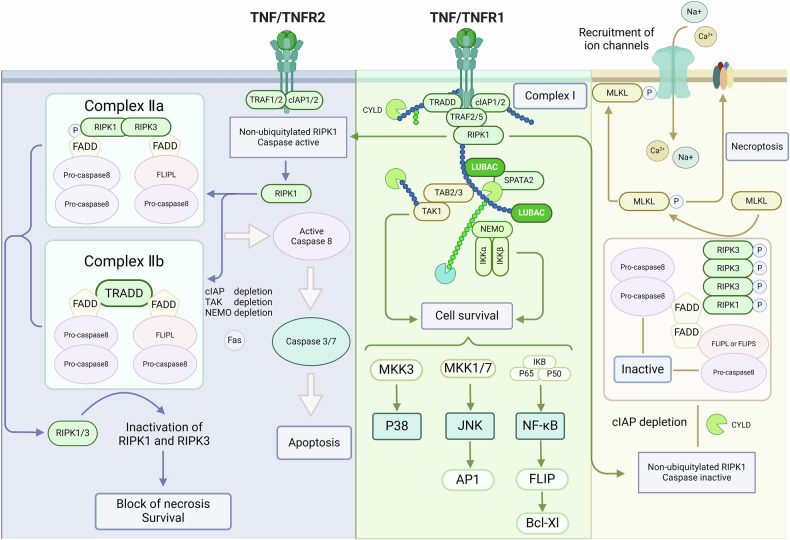
Induced activation of necroptosis pathway via the TNF-α pathway. This is a typical pathway for inducing the occurrence of necroptosis, in which the interaction of death ligands and death receptors further induces the occurrence of a series of cascade reactions downstream.

TNF-α is a pleiotropic proinflammatory factor that acts on the immune system, promotes cell proliferation and differentiation, and regulates cell death [25]. It is produced in activated macrophages as a homotrimeric protein with each subunit containing 157 amino acids. Upon toxicant exposure, TNF-α expression increases, activating its receptors and triggering downstream reactions [26]. TNF-α has two receptors (Fig. 1), TNFR1 and TNFR2. TNFR1, which contains a death domain (DD), is the primary functional receptor, mediating key intracellular activities such as NF-κB activation for cell survival, apoptosis, and necroptosis [27, 28].

TNF-α first binds specifically to the N-terminal ligand-binding pre-aggregation domain of TNFR1, releasing the death domain silicon. TNFR1 binds to a protein that also contains DD through DD to form complex I. These DD-containing proteins include RIP1, TNF receptor-associated domain protein TRADD, FADD, and linear ubiquitin chain assembly complex (LUBAC), and several including TNF-αreceptor associated factor 2/5 (TRAF2/5), an inhibitor of apoptosis proteins (IAPs), cIAP1, cIAP2 E3 ubiquitin ligase [29]. E3 ubiquitin ligases such as TRAF2/5, cIAP1/2, and LUBAC promote the addition of the Lys63 polyubiquitin protein to the Lys377 site of RIP1 to ubiquitinate RIP1 [30–32]. LUBAC ubiquitinates NF-κB essential modifier (NEMO), thereby promoting the activation of NF-κB signaling pathway apoptosis [22]. Deubiquitination of E3 ubiquitin ligase, deletion of LUBAC, or deubiquitination of RIP1 by CYLD in complex I leads to the restriction of NF-κB activation, which promotes the formation of complex IIa and activates caspase-8-dependent apoptosis [22, 30, 33]. RIP1 requires deubiquitination to participate in TNFR1-mediated apoptosis and necroptosis. RIP1 in complex I is released into the cytoplasm after deubiquitination, and TRADD is also released after the internalization of TNFR1 [34]. In the cytoplasm, TRADD, FADD, RIP1, and caspase-8 interact to form complex IIa, also known as death-inducing signaling complex (DISC), which activates caspase-8-mediated apoptosis [35]. Activated caspase-8 can cleave RIP1 and RIP3 (Asp328), resulting in the inhibition of RIP activity [36]. RIP1 cannot reactivate NF-κB, and RIP1 and RIP3 cannot participate in necroptosis [19, 37]. When caspase-8 is depleted or its activity is inhibited, RIP1 and RIP3 kinase domains are activated. RIP3 binds to RIP1, causing autophosphorylation of RIP3 and phosphorylation of RIP1, which binds to MLKL protein downstream of RIP3 to form complex IIb, microsomes. It mediates the necroptosis of cells [7, 38, 39].

### Role of RIPK1 in necroptosis

**Fig. 2:**
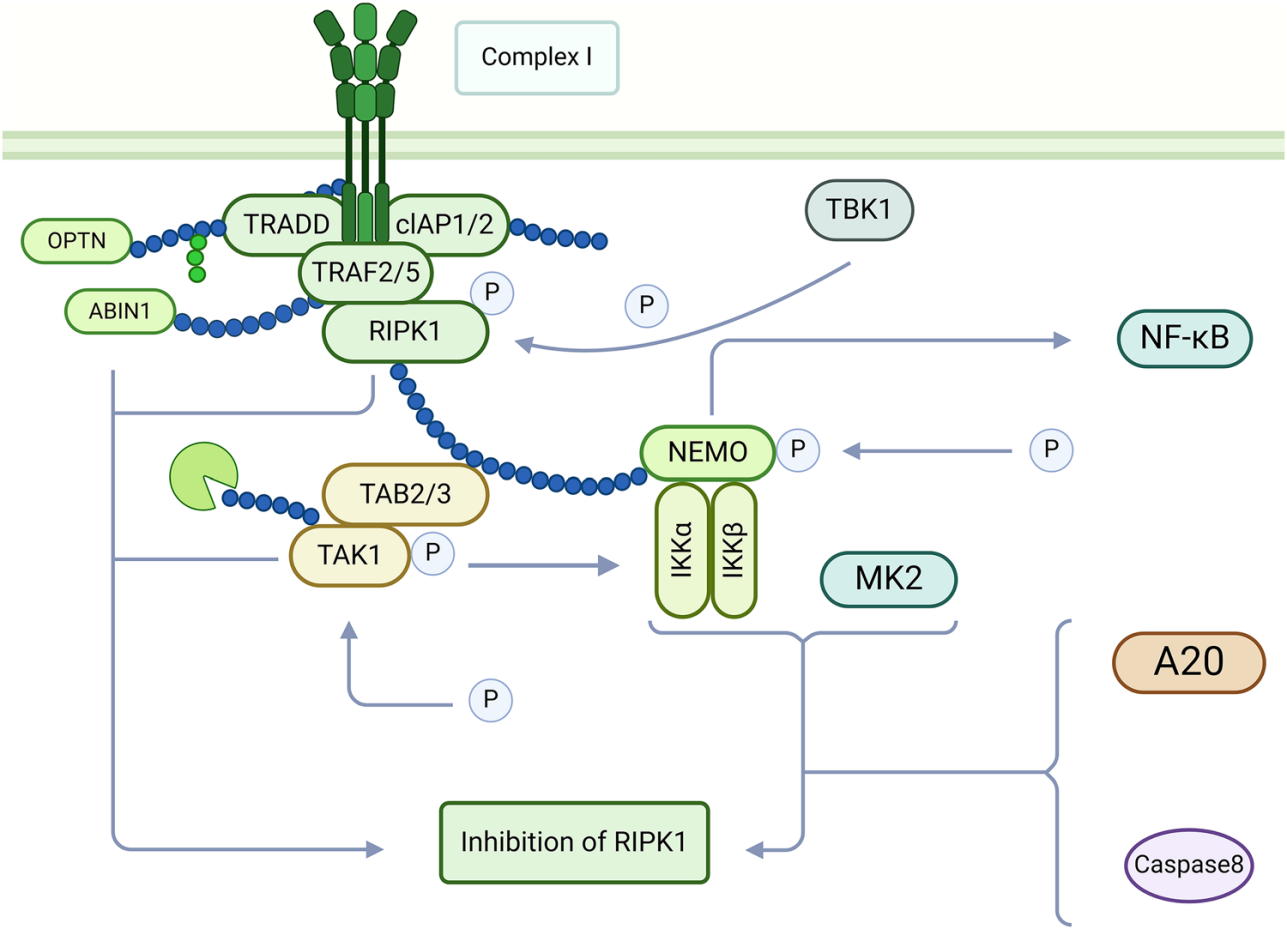
RIPK1 is involved in regulating the occurrence of necroptosis processes. RIPK1 is involved in the signaling of multiple signaling pathways and is even related to survival pathways. In the necroptosis pathway, RIPK1 plays a crucial role, not only in the recruitment of death domains, but also in the formation of necrosome complexes, thereby activating Caspase-dependent and Caspase-independent cell death pathways.

As members of the receptor-interacting protein family, RIP1 and RIP3 both contain a serine/threonine protein kinase domain (KD) at the N-terminus and a RIP homotypic interaction motif (RHIM) in the middle. RIP1 interacts with RIP3 through the RHIM to form an amyloid structure that triggers necroptosis [38]. RIP1 also interacts via the RHIM with other proteins that also contain RHIM domains [40]. RIP1 recruits TLR3 and TLR4 by interacting with the RHIM of TRI and then activates dsRNA in the body, lipopolysaccharide (LPS), or cytoplasmic dsRNA via TLR3, TLR4, and DAI to activate pro-inflammatory mechanisms NF-κB and IRF-dependent signaling respectively [41]. Unlike other members of the RIP family, RIP1 has a death domain at the C-terminus that can bind directly to death receptors (such as TNFR1 and Fas, etc.) or DD-containing adapter proteins (such as TRADD and FADD, etc.), thereby initiating related signaling pathways [42] (Fig. 2). As an important target of cell necroptosis, RIPK1 is also an important pathway for some nanomaterials such as N-TiO_2_ and SeNPs to induce cell necroptosis, which is an important direction in the research of nanomaterials in the treatment of cancer [43, 44]. Studies suggest that RIP1 plays an important role in NF-κB activation, apoptosis, and necroptosis [45]. The ubiquitylation state of RIP1 determines whether RIP1 functions to promote cell survival or to mediate cell death and necroptosis. RIP1 is not required for NF-κB activation, and RIP1 involvement in NF-κB activation and apoptosis depends on the middle RHIM and the C-terminal death domain of RIP1. However, the kinase activity of RIP1 is required to induce necroptosis [46]. The chemical small molecule Nec-1 is now recognized as a specific necroptosis inhibitor, which can specifically inhibit the activity of RIP1 kinase, prevent the interaction between RIP1 and RIP3, and inhibit the phosphorylation level of RIP3, thus effectively reducing the occurrence of cell necroptosis and increasing the cell survival rate. In turn, it can ameliorate renal ischemia and reperfusion injury and retinal ischemia and reperfusion injury [47–49]. Linkermann et al. showed that Nec-1 could inhibit RIP1-mediated necroptosis in renal ischemia-reperfusion injury [50].

### Role of RIPK3 in necroptosis

**Fig. 3:**
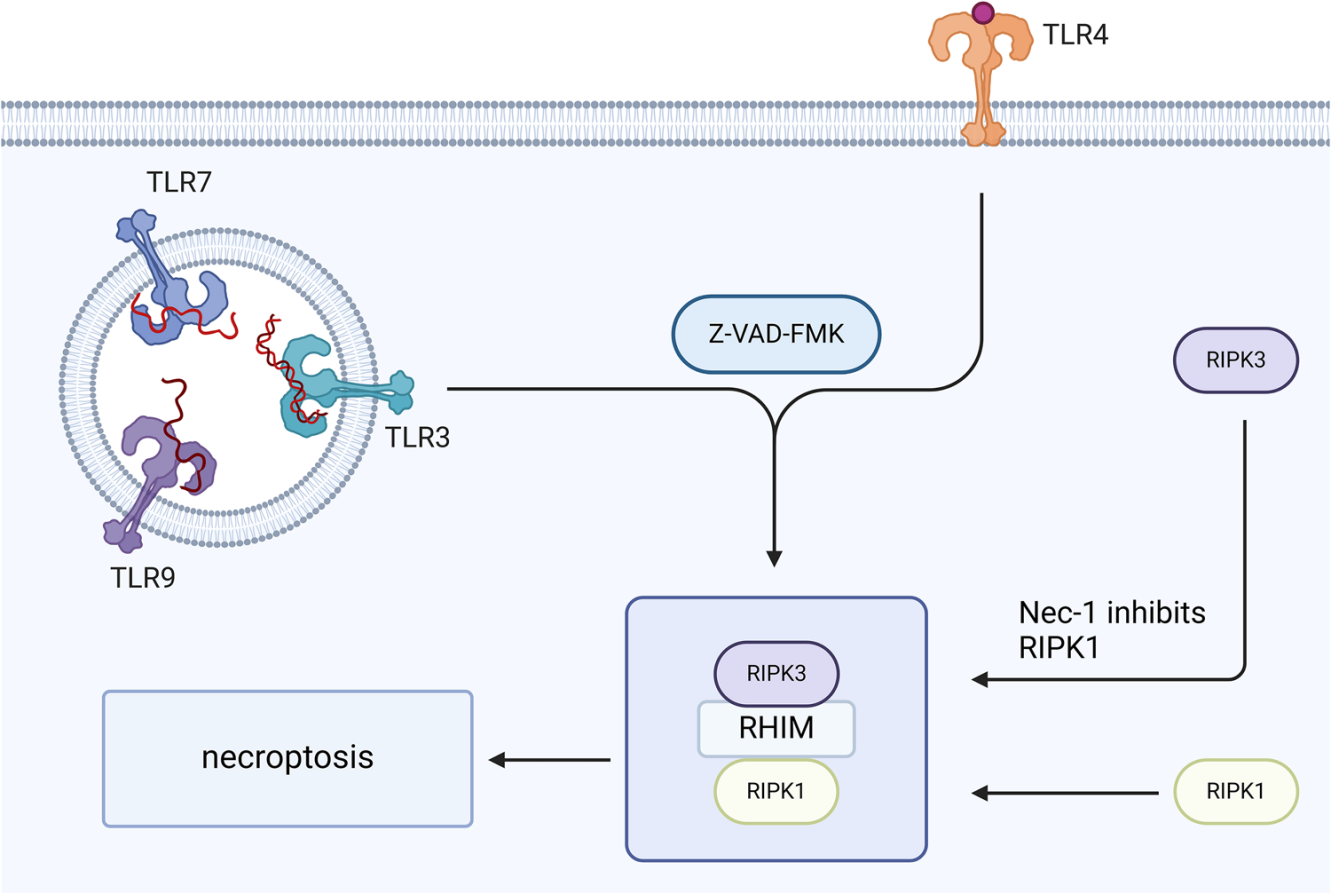
RIPK3 and RIPK1 combine to form necrosomes, forming molecular switches that occur during necroptosis. RIPK3 can be inhibited by its specific inhibitor GSK872, thereby inhibiting the binding of necrosomes, and reducing the occurrence of a series of downstream cascade reactions.

Many studies have confirmed that RIP3 is closely associated with the induction of necroptosis [51, 52]. RIP3 expression is increased in several disease models that induce necroptosis. For example, increased expression of RIP3 was detected in the tissues of azurin-induced pancreatitis, and tissue damage was inhibited after RIP3 deletion [53]. Normally, RIP1 and RIP3 are present in the cytoplasm, but RIP3 was found to be able to penetrate the nuclear membrane into the nucleus [54]. It has been suggested that RIP3 can also mediate the development of necroptosis in the absence of RIP1 [55] (Fig. 3). When ischemia-reperfusion injury occurs, RIP3 expression is upregulated and nuclear translocation occurs in hippocampal CA1 neurons to induce necroptosis, but RIP1 is not affected. In addition, the viral RIP3 inhibitor M45 acts specifically on the RHIM of RIP3 to inhibit the interaction between RIP3 and RIP1, blocking TNF-α-induced necroptosis and RIP3-dependent necroptosis without affecting RIP1 [56]. It is therefore suggested that RIP3 is required for necroptosis, whereas RIP1 may only be involved in necroptosis induced by certain stimuli.

### Role of MLKL in necroptosis

**Fig. 4:**
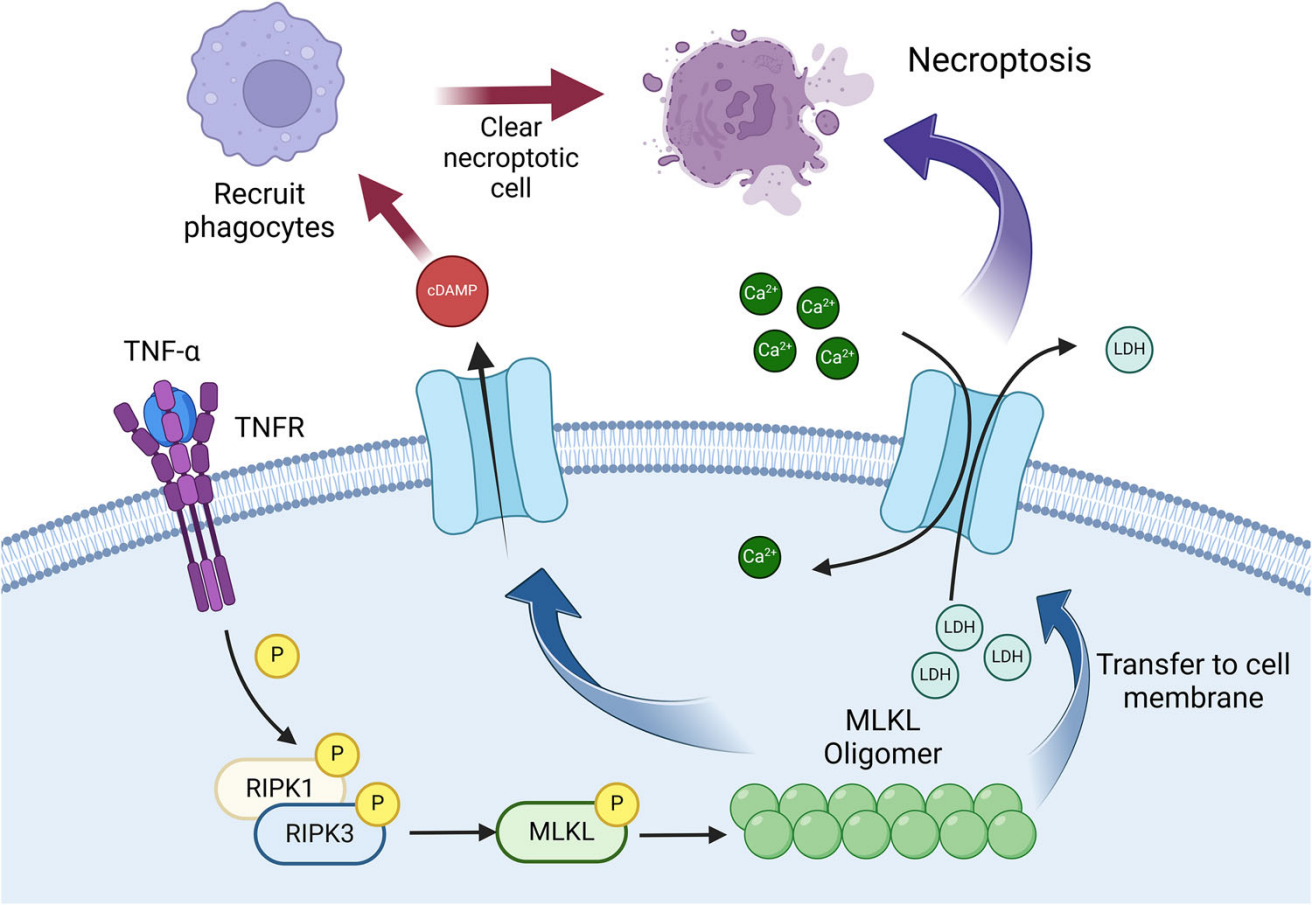
Process of necroptosis resulting from post-translational modification of RIPK1, RIPK3, and MLKL. Upon TNFR activation, RIPK1 binds to RIPK3 to form the necrosome, which subsequently promotes MLKL phosphorylation. Phosphorylated MLKL forms pores in the plasma membrane, leading to Ca^2+^ influx and LDH efflux, ultimately triggering necroptosis. Concurrently, the cell releases damage-associated molecular patterns (DAMPs) to attract phagocytes for the clearance of damaged cells.

MLKL is a key effector protein in the process of necroptosis [57], belongs to the protein kinase superfamily and contains a protein kinase-like domain without catalytic activity, making it a pseudokinase [58]. It comprises a four-helix bundle (4HB) domain and a pseudokinase domain, both of which are involved in its oligomerization and activation [59]. Various extracellular or intracellular signals directly or indirectly activate RIPK3 via RIPK1. RIPK3 and MLKL are essential components of necroptosis [60]. During necroptosis, RIPK3 is phosphorylated at the Ser227 site, which is essential for the activation of MLKL [61]. Phosphorylated MLKL (p-MLKL) is a trigger for necroptosis and is not present in normal tissues or cells but can only be detected in infected, damaged, or aged tissues [62]. The activation of MLKL leads to a conformational switch and translocation from the cytoplasm to the plasma membrane [63]. MLKL is localized in the cytoplasm and translocates to the plasma membrane during necroptosis [64]. In the molecular dynamics mechanism of MLKL interaction with the plasma membrane, the H4 helix region of MLKL first anchors onto the phospholipid membrane after it binds from an aqueous solution [65]. The 4HB domain is inserted into the phospholipid membrane only after the scaffold helix H6 separates from the 4HB domain. Activated MLKL forms pores on the plasma membrane, leading to membrane rupture and leakage of cellular contents, ultimately triggering necroptosis (Fig. 4) [66].

### The clinical significance of developing degraders for RIPK1

Recently, RIPK1 has emerged as a key regulator of cell fate and immune responses, with its roles in various diseases gradually being elucidated, making it a popular target for drug development [67]. RIPK1 is involved not only in cell death processes such as apoptosis and necroptosis but also in the regulation of inflammatory responses and immune reactions [68, 69].

In the research on RIPK1 degraders, the application of proteolysis targeting chimera (PROTAC) technology has provided new insights for RIPK1-targeted therapy [70]. For example, a research team at Baylor College of Medicine developed the RIPK1 degrader LD4172, which achieved specific degradation of RIPK1 using PROTAC technology. Studies have shown that LD4172 exhibits high-efficiency RIPK1 degradation activity both in vitro and in vivo, significantly enhances tumor immune responses, and sensitizes tumors to anti-PD-1 therapy. Moreover, by degrading RIPK1, LD4172 promotes TNF-α-induced apoptosis and immunogenic cell death, thereby remodeling the tumor immune microenvironment [71]. These findings suggest that RIPK1 degraders hold promise as a novel strategy to enhance the efficacy of cancer immunotherapy.

In the clinical research of RIPK1 inhibitors, several RIPK1 inhibitors have entered clinical trial stages. For example, GSK31450595, a highly specific RIPK1 kinase inhibitor, is currently undergoing Phase I clinical trials for pancreatic cancer and other solid tumors. This inhibitor not only demonstrates robust RIPK1 inhibitory activity in vitro but also promotes the formation of tumor-suppressive T cell phenotypes, showing potential antitumor activity [72]. Additionally, the RIPK1 inhibitor GSK2982772, which has been proven to have good kinase specificity and can significantly block TNF-dependent cellular responses, is currently in Phase IIa clinical studies for psoriasis, rheumatoid arthritis, and ulcerative colitis [73].

## Regulatory mechanisms associated with necroptosis

### Modification by ubiquitination

Post-translational protein modifications include phosphorylation, cleavage, ubiquitination, etc. Ubiquitination mediates the covalent attachment of a small 8-KDa ubiquitin protein to the substrate protein, including monoubiquitylation and the formation of different ubiquitin polymers (polyubiquitination) that depend on the activity of three different types of adenosine triphosphate-dependent ubiquitin ligase (E3), ubiquitin-conjugating (E2), and ubiquitin-activating (E1) enzymes [74]. The RIPK1 ubiquitination network controls various signaling processes for protein stability, inflammation, and cell death [75]. The polyubiquitin chains assembled during TNF-α-induced NF-κB and MAPK activation include M1, K11, K48, and K63 ubiquitination, which play a key role in regulating RIPK1 activation [76, 77]. Under normal conditions, RIPK1 activation is inhibited by M1 and K48 ubiquitination. The c-IAP protein is a complex 1-assembled E3 ligase. Linear ubiquitin chains on TRADD and NEMO molecules conjugated to c-IAP1/2 contribute to LUBAC recruitment. This is essential for TNFR1 complex I signal [78]. 71 M1-linked ubiquitin chains were loaded by LUBAC, K377 of RIPK1 was the K63 ubiquitin site after TNF-α stimulation, and linear K-63 ubiquitin chains were added to survival complex I before RIPK1 stabilization [79]. However, K377 is not the only ubiquitination site in RIPK1, and it has never been confirmed by mass spectrometry of endogenous RIPK1. Therefore, some other ubiquitination sites in RIPK1 are very likely to play a role in MAPK and NF-κB activation [75]. TAB2/3 can specifically attach K63-linked ubiquitin chains, whereas NEMO can bind linear, K63-linked, and K11-linked polyubiquitin chains. CYLD restricts NF-κB activation by removing the K63-linked and linear polyubiquitin chains on complex I to switch to cell death signals, whereas A20 binds the linear chains to protect them from cleavage. Ubiquitin thioesterases (OTULIN, Bifidobacterium I protease /FAM105B/Gumby with linear specificity) specifically hydrolyze Met1-linked polyubiquitin chains [80]. XIAP is not associated with complex I, but it inhibits tumor necrosis factor or lipopolysaccharide-induced cell death, and XIAP can indirectly affect RIPK1 ubiquitination by regulating the association of RIPK1 with E3 ligases or deubiquitinases [81]. RIPK1 does not undergo ubiquitination in caspase-8-associated apoptotic complexes. However, certain conditions such as the combination of tumor necrosis factor treatment and TAK1 inhibition can lead to the retention of ubiquitylated RIPK1 in apoptotic complex II, therefore, inhibition of IKKα/IKKβ or tissue-specific NEMO deletion can trigger RIPK1 kinase activity-dependent apoptosis [75, 82, 83]. Different ubiquitin links have been identified in microsomes, suggesting that microsomal RIPK1 (K115) ubiquitination is important for maintaining RIPK1 kinase activity in the microsomal complex, which involves synergistic RIPK1 phosphorylation and ubiquitination [84].

### Modification by phosphorylation

TAK1 can inhibit RIPK1 activation either directly through inhibitory phosphorylation or indirectly through activation of downstream kinases, including MAPK-activated protein kinase 2 and IKKs [82, 85, 86]. Recruitment of NEMO into complex I leads to activation of the IKK complex, which in turn activates NF-κB and inhibits RIPK1 activation through inhibitory phosphorylation [87]. In addition, M1 ubiquitination of RIPK1 in complex I also mediate the recruitment of the kinase TBK1, which leads to the phosphorylation of T189, an important site for RIPK1 substrate recognition, blocking the phosphorylation of RIPK1 itself and preventing its activation [88]. A20 is a ubiquitin processing enzyme that inhibits RIPK1 activation, and A20 contains an I domain that is functionally equivalent to the K63 bisphosphatase of RIPK1. Thus, A20 deficiency activates RIPK1 and RIPK3, thereby promoting necroptosis [89, 90]. Three ubiquitin-binding proteins such as OPTN, NEMO, and ABIN1, which primarily bind to the M1 ubiquitin chain, all negatively regulate RIPK1 activation [77, 91, 92]. Caspase-8 is the major protein that regulates necroptosis and inhibits necroptosis by proteolytically cleaving RIPK1 at Asp324, dissociating the N-terminal kinase domain from the C-terminal portion of the molecule and preventing RIPK1 kinase activation by dimerization via the C-terminal DD [93].

### Mechanism of MLKL regulation

RIPK3 can promote the phosphorylation of MLKL at threonine and serine sites including S158, S124, S228, and S248. Normally, activated RIPK3 interacts with MLKL in the cytoplasm through its C-terminal kinase domain and induces the phosphorylation of MLKL at threonine 357 and serine 358, leading to MLKL oligomerization and membrane translocation of MLKL oligomers [94, 95]. Upon activation, MLKL reveals its N-terminal 4-helix bundle (NB) domain and a central support region, thereby forming a polymer and enhancing its ability to bind PIP (phosphatidylinositol 4, 5-diphosphate is the preferred interacting partner) to the inner leaflet of the plasma membrane, which stabilizes the translocation and binding of the support domain. This initial binding is mediated by the support domain, which has a relatively low affinity but is essential for MLKL translocation to the plasma membrane [96]. The exact stoichiometry of MLKL oligomers has not been elucidated, but it appears that the transport of MLKL oligomers to the plasma membrane is dependent on class A member 1 of the 90 kDa family of cytoplasmic heat shock proteins, such as HSP90 [97]. MLKL oligomers then disrupt membrane integrity and increase membrane permeability through non-specific pore formation or interaction with TRPM7 calcium channel proteins. Activated MLKL is also transported to the inner membrane, increasing organelle permeability, and forming transmembrane cation channels such as Mg^2+^, Na^+^, and K^+^. MLKL-induced membrane depolarization and cell death are triggered [64, 98]. It has been shown that CIAP-mediated ubiquitination of TRAF2 leads to the dissociation of MLKL from necrosomes and that CYLD is able to disrupt the TRAF2-MLKL interaction, suggesting that CYLD and TRAF2, also key initiator molecules, determine necroptosis by controlling MLKL localization in cells [99, 100]. Another related protein that activates MLKL microsomes is PGAM5, which activates the GTPase activity of dynamin-related protein 1 (DRP1), leading to DRP1 dimerization and mitochondrial fission [101]. PELI1 mediates the K63 ubiquitination of RIPK1 in complex IIb and promotes the activation of RIPK3 and MLKL to mediate necroptosis. Cell rupture following necrosis leads to the release of molecular patterns associated with inflammatory damage, which can activate the innate immune system and neighboring cells via PRRs [84, 102]. Polyubiquitination of MLKL and RIPK3 in microbodies may be mediated by TLR signaling, the role of the modification is not known and the relevant E3 ligases have not been identified, future studies are clearly needed to elucidate the functional role of MLKL and RIPK1/3 polyubiquitination in necroptosis [103].

## Necroptosis and tumor

Necroptosis plays an important role in maintaining tissue homeostasis. Known mode of cell death mainly includes apoptosis, necroptosis, pyroptosis, ferroptosis, and so on [104]. Apoptosis is a natural barrier to preventing cancer and is also an important mechanism for anti-tumor drugs. Apoptosis tolerance can promote the occurrence, development, and drug resistance of tumors, leading to the failure of tumor treatment. When the apoptotic mechanism is impaired, necroptosis acts as another barrier to inhibit tumor development, which can effectively prevent tumor progression [16]. The important role of necroptosis in tumor biology makes it a new target for tumor therapy, and an increasing number of compounds and drugs inhibit tumor development by inducing necroptosis.

Necroptosis has a dual role in promoting and inhibiting tumor growth. In cells that have failed to induce apoptosis, necroptosis can be used as an alternative cell death pathway to prevent tumor development. However, as a mode of death that induces cell necrosis, necroptosis can trigger an inflammatory response, which in turn promotes tumorigenesis and metastasis [105, 106]. Induction of necroptosis can inhibit tumor development. Downregulation of many key molecules in the necroptosis pathway has been found in various types of cancer cells, suggesting that cancer cells may survive by evading necrosis [107–109]. RIPK3 expression is absent or reduced in many cancer cell lines [110, 111]. In patients with ovarian cancer, pancreatic cancer, and cervical squamous cell carcinoma, low levels of MLKL are associated with reduced overall survival [112–114]. These findings suggest that RIPK3 and MLKL are important tumor suppressors and are closely related to tumor prognosis. Based on these studies, activating or restoring the necroptosis-related signaling pathway, may be used as a method to treat cancer. On the other hand, necroptosis can also promote tumor initiation and metastasis. Strilic et al. found that mouse and human lung cancer cells can induce necroptosis of endothelial cells and promote tumor cell escape and metastasis [115]. Seifert et al. reported that necroptosis promotes pancreatic cancer development, found that RIPK1 and RIPK3 were highly expressed in pancreatic ductal adenocarcinoma, and inhibition of RIPK1 or deletion of RIPK3 prevented pancreatic cancer progression in vivo [116]. Liu et al. reported that silencing RIPK1 or RIPK3, key regulators of necroptosis, in cancer cells significantly attenuated cancer cell proliferation and reduced the ability to form tumors in vivo [117]. Given the dual role of necroptosis in tumorigenesis and development, further research into the molecular mechanism of the beneficial anti-tumor effect of necroptosis may maximize the anti-tumor effect of necroptosis [118].

## Nanoparticles—as therapeutic agents

Although traditional treatments such as chemotherapy and radiotherapy can control tumor growth to some extent, they often damage healthy cells and cause various side effects in patients. Therefore, the development of a treatment that can not only accurately identify cancer cells but also effectively kill cancer cells has become a research hotspot in the field of cancer treatment. Nanomaterials have been widely studied as an emerging therapeutic approach [119]. However, with the development of the field of nanomedicine, people have questioned the potential impact and unpredictable adverse effects of the application of nanomaterials in cancer treatment [120]. To date, the cytotoxic effects of nanomaterials have been studied based on cell survival and cell functions, such as membrane integrity, mitochondrial activity, and cell morphology. It is increasingly recognized that a more detailed analysis of nanomaterial-induced RCD is essential to understand the full mechanism of action. Such knowledge will help us to design safe therapies and increase the therapeutic potential of anticancer drugs.

In recent years, an increasing number of studies have shown that there is a close relationship between necroptosis and nanomedicine, which is a new strategy for the development of nanomaterial-based therapeutic agents for the efficient treatment of various cancers. These nanomaterials include liposoluble nanomaterials and nanomaterials with targeting properties [120]. On the one hand, these nanomaterials are endowed with the ability of cellular uptake to enhance the accumulation of ROS, which ultimately leads to cell death. On the other hand, due to their nanoscale size, engineered nanomaterials tend to passively target tumor tissues through enhanced permeability and retention (EPR) effects, leading to cancer-specific therapies [121].

### Characteristics of nanomaterials

The importance of nanomaterials in necroptosis-based therapy stems from their unique physicochemical properties and extensive potential for biological applications [122]. Nanomaterials typically refer to materials with sizes ranging from 1 to 100 nanometers, whose small size confers distinct characteristics different from macroscopic materials, enabling them to play a crucial role in tumor therapy, particularly in inducing and regulating necroptosis [123]. The size effect of nanomaterials increases their surface-to-volume ratio, significantly enhancing chemical reactivity, which is critical for drug release and intracellular signal transduction, thereby improving drug bioavailability and facilitating rapid entry into target cells [124]. Additionally, the surface of nanomaterials can be chemically modified to enhance biocompatibility and targeting ability, for instance, by conjugating antibodies, ligands, or small molecules to achieve targeted recognition and binding to specific tumor cells, thereby improving the specificity and efficacy of treatment [125–127]. Some nanomaterials, such as quantum dots or metallic nanoparticles, possess excellent optical properties that can be used for imaging and monitoring [128, 129]. Fluorescent nanomaterials can track drug distribution in real-time within the body, while magnetic nanomaterials can achieve targeted localization and control via external magnetic fields [130, 131]. The multifunctionality of nanomaterials allows them to be designed as multifunctional carriers, capable of delivering therapeutic drugs while simultaneously carrying imaging agents, diagnostic molecules, etc., enabling concurrent therapy and monitoring to enhance overall therapeutic outcomes [132].

### Regulatory role of nanomaterials in necroptosis

Nanomaterials are increasingly being used in the treatment of cancer, and they play different roles in the treatment of cancer through different pathways [123] (Table 1). In the process of treatment, some nanomaterials themselves can play a role in regulating the necroptosis pathway in cancer cells to induce necroptosis of cancer cells and then treat cancer. Se nanoparticles are considered the most promising nano systems due to their high anticancer activity and good biocompatibility. Praveen Sonkusre et al. found that exposure of PC-3 cells to SeNPs activated TNF and IRF1, while SeNPs were internalized by the cells and induced mitochondrial ROS production. The activation of TNF and IRF1 genes and the generation of ROS are involved in the necroptosis pathway leading to cell death. This cellular necroptosis pathway is dependent on RIP1 and independent of RIP3 and MLKL [44]. Other nanomaterials can be directly targeted to attack cancer cells by coupling with drugs or acting as drug carriers, which are characterized by efficient treatment with low doses of drugs while greatly reducing systemic toxicity and carrying drug molecules with longer half-lives. Xiao et al. found that overexpression of the proto-oncogene HER2 in unresectable or recurrent osteosarcoma can be used as a therapeutic target. The anti-HER2 antibody trastuzumab (TRA) had no significant effect on OS, but the TRA/GO complex formed after non-covalent binding to the nanomaterial graphene oxide (GO) caused the immediate degradation of IAP (a cellular inhibitor of apoptotic proteins) and caspase 8 by inducing oxidative stress and strong HER2 signaling. Activation of necroptosis leads to rapid death and kills OS cells [133]. The combination of nanomaterials with ultrasound or phototherapy is also one of the commonly used feasible treatments. Zhang et al. designed a novel biomimetic nanoparticle to deliver interleukin-12 messenger RNA using crGD-modified cancer cell membranes (CM) coated with calcium carbonate nanoparticles. The crGD-modified CM provided the nanoparticles with BBB cross-over and tumor homing/isotype targeting capabilities. The CaCO_3_NPs core exposed to ultrasound (US) can induce cell necroptosis through the CO_2_ bubble-mediated cavitation effect, leading to DAMPs release and DC maturation. Simultaneous binding of IL-12 mRNA induced excellent antitumor activity against GBM both in vitro and in vivo [109]. Chen et al. found that CuS-MnS_2_ nanoflowers exhibited high photothermal conversion efficiency and simultaneous ROS generation under NIR irradiation, and the combination of CuS-MnS_2_ and 808 nm NIR laser showed good tumor ablation effect through the necroptotic pathway. It is a promising phototherapeutic agent for cancer [134].

**Table 1:** Nanomaterials that induce necroptosis through different pathways.

Types	Nanomaterials	Cell types	Reference
Promote necroptosis	SeNPs	Prostate adenocarcinoma cells	[44]
Drug coupling	GO	Unresectable or recurrent osteosarcoma cells	[133]
	AuNPs	Chronic myeloid leukemia cells	[146]
	GO	Non-small cell lung cancer cells	[147]
Combined with ultrasound	Calcium carbonate nanoparticle	Glioblastoma	[109]
Combined with phototherapy	CuS-MnS_2_	Human ovarian carcinoma cell	[134]
	CuS-NiS_2_	Human gastric cancer cells	[148]

This table summarizes the mechanisms by which various nanomaterials induce necroptosis in cancer cells, including the types of nanomaterials, pathways involved, and the cancer cell types studied.

In summary, nanomaterials can induce necroptosis to treat diseases and can be combined with the same treatment to improve the therapeutic effect, showing great potential in the treatment of diseases.

### Safety issues of nanomaterials

The safety of nanomaterials used to treat disease should also be considered. Nanomaterials can be induced to treat diseases such as cancer through the necroptosis pathway, but at the same time the use of nanomaterials can also lead to some other injuries [135].

Due to the complex behavior of nanomaterials in vivo, it is necessary to evaluate their long-term biocompatibility and potential toxicity. The behavioral differences of various types of nanomaterials in the body may lead to different biocompatibility issues [136]. Additionally, their high surface-to-volume ratio allows nanomaterials to interact more closely with endogenous molecules, potentially causing cytotoxicity and tissue damage, which requires systematic evaluation through in vitro experiments [137, 138]. The distribution, metabolism, and excretion of nanomaterials in the body are key factors affecting their biosafety, as long-term accumulation may lead to tissue toxicity or functional impairment [139, 140]. Furthermore, issues related to immune responses cannot be ignored, as nanomaterials of different sizes, shapes, and surface modifications can stimulate the immune system to varying degrees. As an inorganic compound, TiO_2_ has been used in photodynamic therapy for various diseases due to its ability to generate ROS to induce cell death. However, studies have shown that alveolar macrophages engulfing TiO_2_ nanoparticles show obvious characteristics of necrotizing apoptosis, and necrotic alveolar macrophages can lead to the occurrence of lung inflammation [141]. ZnO nanoparticles have great potential in drug delivery due to their excellent biocompatibility and low cost. Currently, many drugs use ZnO as a delivery agent. Some studies have shown that ZnO nanoparticles can trigger the production of many intracellular ROS by releasing free Zn^2+^, which leads to the autophagic death of immune cells. This impairs host immunity [142].

Nanomaterials have demonstrated significant potential in regulating necroptosis for tumor treatment, but their biosafety issues cannot be overlooked. Future research needs to delve deeper into exploring safer and more efficient applications of nanomaterials to achieve better clinical outcomes.

### Prospects of nanomaterials in necroptosis-based therapy

Necroptosis is closely related to the occurrence and progression of various tumors and is considered an important target for cancer therapy due to its unique cell death mechanism [16]. Nanomaterials, with their size effect, surface modification capabilities, and functional characteristics, show great potential in tumor treatment. By precisely designing nanomaterials, it is possible to effectively target necroptosis-related sites. The potential advantages of nanomaterials are evident in several areas: functionalized nanocarriers can significantly enhance drug accumulation within tumor cells and improve the therapeutic efficiency of necroptosis inducers, while accurately delivering drugs to targets to reduce toxicity and side effects on normal cells [143]. Moreover, the multifunctional characteristics of nanomaterials allow for the combination of multiple therapeutic approaches to achieve synergistic effects and enhance overall therapeutic outcomes [144]. In the future, treatments based on nanomaterials should focus on improving their targeting of tumor cells and tailoring personalized treatment plans based on individual patient differences. Developing smart nanomaterials that respond to changes in the tumor microenvironment will enable more precise drug release and therapeutic control, while combining nanomaterials with other treatment modalities to explore their potential applications in multimodal therapy [145].

## Conclusion

In this review, we have mainly investigated the regulatory mechanisms of necroptosis, the roles of some related regulatory factors in necroptosis, and different induction modes of necroptosis. At the same time, we also discuss the recent research progress of nano-induced necroptosis for cancer treatment. This is a promising area of research, and some progress has been made, but there is still a lot of work to be done, including safety and feasibility. In the future, it is hoped that this treatment will play a greater role in the treatment of cancer and a wide range of diseases, bringing hope and rehabilitation to more patients. We hope that this review will be useful for future studies on nanomaterial-induced necroptosis.
